# Higher pulmonary vein index on computed angiography and optimum surgical resection ensures smooth postoperative recovery in Fallot's tetralogy: Special emphasis on indices of evaluation and Monocusp preparation

**DOI:** 10.1002/ccr3.6100

**Published:** 2022-07-27

**Authors:** Vishal V. Bhende, Tanishq S. Sharma, Deepakkumar V. Mehta, Krishnan Ganapathy Subramaniam, Amit Kumar, Jigar P. Thacker, Viral B. Patel, Gurpreet Panesar, Kunal Soni, Kartik B. Dhami, Hardil P. Majmudar, Nirja Patel, Sohilkhan R. Pathan

**Affiliations:** ^1^ Pediatric Cardiac Surgery, Bhanubhai and Madhuben Patel Cardiac Centre Shree Krishna Hospital, Bhaikaka University Karamsad India; ^2^ Department of Radiodiagnosis & Imaging Pramukhswami Medical College & Shree Krishna Hospital, Bhaikaka University Karamsad India; ^3^ Cardiac Surgery MGM Healthcare Chennai India; ^4^ Pediatric Cardiac Intensive Care Bhanubhai and Madhuben Patel Cardiac Centre Shree Krishna Hospital, Bhaikaka University Karamsad India; ^5^ Department of Pediatrics Pramukhswami Medical College, Bhaikaka University Karamsad India; ^6^ Cardiac Anaesthesiology, Bhanubhai and Madhuben Patel Cardiac Centre Shree Krishna Hospital, Bhaikaka University Karamsad India; ^7^ Clinical Research Services Bhanubhai and Madhuben Patel Cardiac Centre Shree Krishna Hospital, Bhaikaka University Karamsad India

**Keywords:** congenital heart disease, early outcomes, McGoon ratio, pulmonary vein index, tetralogy of Fallot

## Abstract

Tetralogy of Fallot (TOF) is a common cyanotic congenital heart disease. Its surgical correction requires ventricular septal defect (VSD) closure and right ventricular outflow tract obstruction (RVOTO) relief, with transannular patch enlargement (TAPE) of the pulmonary valve. The first successful repair of TOF was reported in 1954 and consisted of closure of the VSD through a large right ventriculotomy, and RVOTO relief with TAPE of the pulmonary valve. To predict the intraoperative requirements and postoperative course of patients with this condition, various evaluation indices are available that can provide a good indication of patient prognosis. We performed this study in a male child (age, 1 year, 9 months; weight 8.5 kgs.) who underwent intracardiac repair for TOF as a primary procedure. We calculated the pulmonary vein index (PVI), McGoon ratio, and Nakata index. The McGoon ratio was 1.97, Nakata index was 539.22 mm^2^/m^2^, and PVI was 368.12 mm^2^/m^2^. The child had an uneventful post‐operative course with no symptoms of low cardiac output syndrome. He was ventilated for 122 h. The length of intensive care unit and hospital stays were 11 and 14 days, respectively. The PVI is a novel indicator offering prognostic indications for pediatric cardiac patients who have undergone surgical correction of TOF.

## Graphical abstract

Correction of TOF is associated with a low mortality risk (1%–2%). The McGoon ratio and Nakata index are based on the size of the pulmonary artery and correlate well with mortality risk. However, another parameter is required to predict morbidity and length of hospital stay. The pulmonary vein index (PVI), which is the total cross‐sectional areas of the pulmonary veins after the entry of all venous branches indexed to body surface area(cutoff >300 mm^2^/m^2^), can be used as a better predictor of pulmonary flow, early postoperative morbidity, and length of hospital stay. The creation of a tailored monocusp, depending on the dimensions of the right ventricular outflow tract in individual patients, can also help in early postoperative recovery. Further studies are required to determine if we can rely on the automated estimation of the PVI as a prognostic indicator not only for TOF but also for other left‐to‐right shunt lesions.

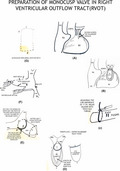

## 1 INTRODUCTION

Tetralogy of Fallot (TOF) is a cyanotic congenital heart disease (CHD) seen in pediatric patients. Successful surgical correction of TOF was first performed in 1954 with the closure of the ventricular septal defect (VSD) through a large right ventriculotomy and relief of right ventricular outflow tract obstruction (RVOTO) by transannular patch enlargement (TAPE).^1^, ^2^

Since then, treatments for the correction of TOF have enjoyed tremendous success.^3^, ^4^ The perioperative mortality rate has reduced to 1.5%, and there are good long‐term outcomes due to the evolution of cardiopulmonary bypass techniques and perioperative management. Although TOF can be lethal if untreated or incorrectly treated; it can have an excellent prognosis with timely surgical intervention (Table 1).

**TABLE 1:** Benefits of early complete repair of TOF

Growth activity of body and organs
Reducing hypoxemia
Limited right ventricular muscle excision
Preservation of left ventricular function
Reduced incidence of late dysrhythmias

The ideal TOF repair removes RVOTO sufficiently to prevent the progression of right ventricular hypertrophy. However, there are remarkable clinical differences between TOF patients, which may lead to overconfidence when formulating treatment decisions and predicting prognoses. TOF patients can experience prolonged postoperative recovery even when surgery is successful.^5^, ^6^

Reduced pulmonary blood flow (Qp) is the main cause of hemodynamic changes in TOF. Presently, preoperative evaluations of TOF children are performed using the McGoon ratio and the Nakata index but these have limitations. Both of these systems measure branch pulmonary arteries (PAs), which can yield fallacious results as pulmonary arteries are not typically cylindrical. Differences may be present as normal anatomic variations or as a result of factors that may increase the stiffness of the vessels^7^, ^8^; therefore, affecting the ability of the PAs to alter their shape in response to changing conditions.^8^ In addition, post‐stenotic dilatation of branch PAs, the presence of major aortopulmonary collateral arteries (MAPCAs), and malformations may lead to misjudgments of Qp and further influence the mis‐estimation of the patient's condition and the determination of poor treatment decisions. Therefore, understanding the anatomy of the pulmonary artery is essential for decision making in pediatric cardiac surgery.^9^

In this study, we aim to demonstrate that the sizes of individual pulmonary veins (PVs) are a more accurate and sensitive indicator of Qp than PA size, especially in TOF patients^10^, ^11^ The pulmonary vein index (PVI) is a new indicator based on the morphology of pulmonary veins. As Qp and the severity of TOF are inversely proportional, PVI may provide more precise predictions.

We review the case of a TOF patient who underwent complete repair in our institution and compare the prognostic abilities of PVI and the McGoon ratio and Nakata index in their predictions of early postoperative TOF outcomes.

## 2 CASE PRESENTATION

Our study was approved by the Institutional Ethics Committee (IEC‐2) of the HM Patel Centre for Medical Care and Education, Anand, Gujarat (approval No.IEC/BU/2021/Cr.54/296, November 27, 2021). Written informed consent to use the patient's data in this study was obtained from a parent or guardian prior to surgery.

The patient was male; aged 1 year, 9 months, 23 days; and weighed 8.5 kg. The weight at birth was 2.5 kg. The patient presented with TOF and confluent branch PAs with a history of cyanotic spells. At the time of surgery, the patient's preoperative oxygen saturation was 75%. The patient had never undergone a surgical procedure prior to this. The McGoon ratio was 1.97, Nakata index was 539.22 mm^2^/m^2^, and PVI was 368.12 mm^2^/m^2^. The operative data comprised a cardiopulmonary bypass time of 152 minutes. The surgical strategy included a combination of four procedures: VSD closure, right ventricular outflow tract (RVOT) resection, TAPE, and atrial septal defect closure. Table 2 presents a summary of the patient information.

**TABLE 2:** Patient information

Basic characteristics	
Age, years	1 year, 9 months, 23 days
Weight, kgs.	8.5 kgs.
Sex	Male
Birth Weight	2.5 kgs.
Pre‐operative oxygen saturation	75%
Cyanotic Spells	+
Previous Surgical Procedure	Nil
McGoon ratio	1.97
Nakata index	539.22 mm^2^/m^2^
Pulmonary vein index	368.12 mm^2^/m^2^
Operative data	
CPB time	196 Min.
ACC time	152 Min.
Surgical strategy	
With TAP	+
Associated procedures	
ASD closure	+

Abbreviations: ACC, aortic cross‐clamp; ASD, atrial septal defect; CPB, cardiopulmonary bypass; PVI, pulmonary vein index; pRV/LV, postoperative right and left ventricle pressure ratio; TAP, transannular patch.

**FIGURE 1:**
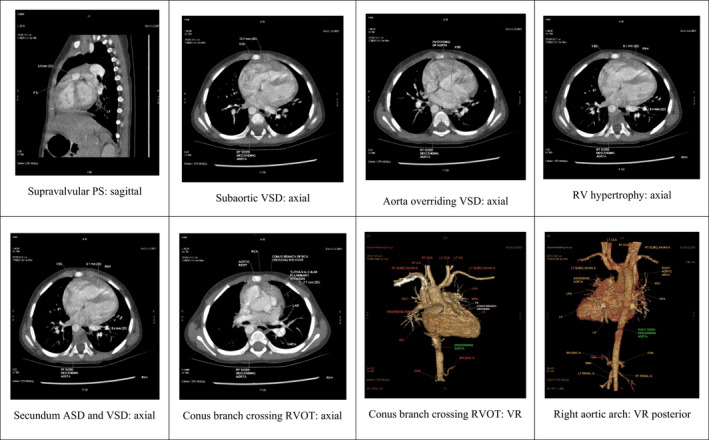
Cardiac CT scan of 1‐ year‐old male child, having Pentalogy of Fallot, right aortic arch, conus branch of right coronary artery crossing anterior to right ventricular outflow tract [RVOT] to left side with Pulmonary Vein Index [PVI] of 368.12 mm^2^/m^2^, McGoon Ratio of 1.97, and Nakata Index of 539.22 mm^2^/m^2^

A cardiac computed tomography (CT) dynamic study revealed confluent branch PAs in our patient. The measurements for these were as follows: right pulmonary artery: proximal 9 mm, distal 8.3 mm; left pulmonary artery: proximal 11 mm, distal 12 mm; main pulmonary artery: proximal (supra‐valvular region) 7.7 mm, mid 9 mm, distal 12.2 mm. (Figure 1).

Pulmonary blood flow has a major effect on the prognosis of patients with cyanotic CHDs. The PA measurements and calculations used in the McGoon ratio and Nakata index are the most commonly used parameters for the evaluation of pulmonary blood flow. Pulmonary artery parameters are widely used prognostic indicators for the successful surgical repair of TOF. (Table 3).

**TABLE 3:** Indices used in the evaluation of TOF patients

Sr.No.	Indices	Description
**Preoperative**		
1	Pulmonary vein index(PVI)^12^	PVI = CSA of Rt. SPV + CSA of Rt. IPV + CSA of Lt. SPV + CSA of Lt. IPV (mm^2^)/body surface area (BSA) (m^2^) Interpretation: PVI <300 mm^2^/m^2^: Delayed Post‐operative Recovery
2	Mc Goon ratio	This is the sum of diameters of immediately pre‐branching left and right pulmonary arteries to the descending aorta just above the level of the diaphragm. The normal value is >2–2.5
3	Nakata index	The Nakata index is a cross‐sectional area of the left and right pulmonary artery in mm^2^ divided by total BSA. The cross‐sectional area is measured by (π × 2/diameter × magnification coefficient expressed for BSA). The cross‐sectional area is calculated by using the formula π × r^2^ × magnification coefficient, where r = radius or 1/2 of the measured PA diameters. A normal Nakata value is 330 ± 30 mm^2^/BSA
4	Total neopulmonary artery index (TNPAI)	Suggested as an indicator of postoperative RV/LV pressure ratio. The TNPAI is calculated by determining the cross‐sectional area of the PAs (as described by Nakata et al.) and the MAPCAs and dividing the result by body surface area (mm^2^/m^2^). TNPAI = APC index + Nakata index/BSA where the APC index is the sum of the CSAs of all usable APCs APC = aorto‐pulmonary collaterals; CSA = cross‐sectional area
5	Z score (Established by Rowlatt, Rinoldi, and Lev)^13^	Observed value–Expected value/Standard deviation (SD) of the expected value
6	Pulmonary annulus index (PAI)^14^	Actual pulmonary annulus diameter(PAD_A_)/ Expected pulmonary annulus diameter(PAD_E_) = PAI PAD_E_ = PAD_A_ + AAD/2 where, AAD = aortic annulus diameter
7	Transannular patch enlargement (TAPE) of the pulmonary valve^15^, ^16^, ^17^	Pulmonary valve annulus(PVA) size /aortic valve annulus (AVA) size TAPE = PVA/AVA Or Great artery annulus size ratio (GA ratio) Many centers use the PVA Z score to determine whether to apply the TAPE procedure, but the cut off value for this measure varies among studies. Choi et al.^15^ demonstrated a cut off value for the z score as −1.67. Stewart and associates^16^ demonstrated that a favorable z score was −4, whereas Awori and colleagues^17^ recommended TAPE when the z score was significantly smaller than −1.3
**Intra‐operative:** pRV: LV ratio {Kirklin's index} < 0.7 = optimal; >0.8 = TAPE		
**Postoperative**		
8	Tricuspid annular plane systolic excursion(TAPSE)	TAPSE is another two‐dimensional measure with which one can assess systolic right ventricular function. TAPSE in pediatric patients. Normal >12 mm Mild RV dysfunction 10–12 mm Moderate RV dysfunction 8–10 mm Severe RV dysfunction <7 mm
9	Fractional area change(FAC)	The fractional area change is a two‐dimensional measure of right ventricular global systolic function. RV FAC (%) Reference range 32%–60% Mild abnormal 25%–31% Moderate abnormal 18%–24% Severe abnormal <17%
10	Pulmonary regurgitation (PR) in postoperative TOF for determining right ventricular contractile dysfunction^18^	PR is classified as follows: Mild (No retrograde diastolic flow in the pulmonary trunk with a detectable regurgitant jet in the RV outflow tract) Moderate (retrograde diastolic flow in the main pulmonary artery) Severe (additional retrograde diastolic flow in branch PAs)
11	Isovolumic myocardial acceleration(IVA)	Experimental and a clinical study as a new tissue Doppler‐based index of systolic RV function post‐TOF repair. IVA demonstrates reduced contractile function in relation to the degree of PR and may be an early, sensitive index for selecting patients for valve replacement.
12	Pulmonary Regurgitation(PR) used to calculate pulmonary artery diastolic pressure (using the modified Bernoulli equation) and mean pulmonary artery pressure	PADP = RVEDP + Î′′ Ppv where PADP = Pulmonary artery diastolic pressure RVEDP = Right ventricular diastolic pressure Î′′ Ppv = Equals the pressure gradient between the pulmonary artery and right ventricular outflow tract. Calculate the end‐diastolic gradient between the pulmonary artery and right ventricular outflow tract from the velocity of the PR jet. Add, assumed RVDP = RAP; then the equation can be applied: PADP = RVEDP + Î′′ Ppv PA_mean_ = (PA_Systolic_ + 2 PA_diastolic_)/3

A previous study by Yuan et al.^12^ evaluated the predictive abilities of the PVI for the early outcomes of TOF repair. They concluded that cardiac CT scans provide the fastest means of gathering the relevant measures compared with echocardiography and magnetic resonance angiography and provide better spatial resolution. Cardiac CT scans are limited by neither the small acoustic windows found in echocardiography nor by motion artifacts in patients with high heart rates, long scan times, or the need for sedation or anesthesia in magnetic resonance angiography. Hence, the authors of that study recommended cardiac CT scans as the gold standard modality for the assessment of pulmonary vein morphology and measurements.

**FIGURE 2:**
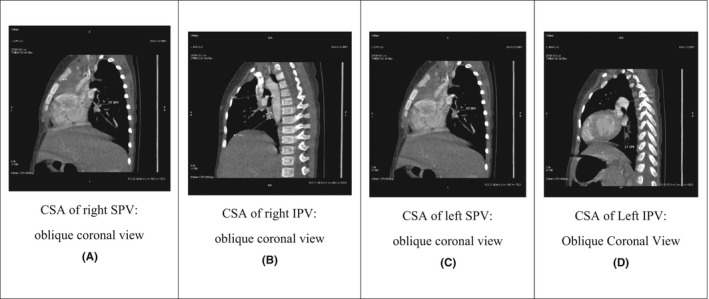
Cardiac CT scan of 1year‐old male child, having Pentalogy of Fallot and right aortic arch (same patient, whose images are shown in Figure 1). Measurements of cross‐sectional area (CSA) of all four pulmonary veins (PV) were taken perpendicular to their lumen about 3 mm before it drains into the left atrium in the oblique coronary images. This patient has a pulmonary vein index (PVI)of 368.12 mm^2^/m^2^

In the past, many researchers have measured the diameters of the PVs just proximal to their ostia in the left atrium and then divided the measurement by 2 to obtain the radius of the lumen. They then calculated the cross‐sectional area (CSA) using the formula for determining a circular area (Figure 2A–D). However, the CSA of a pulmonary vein is not always circular.

For the PVI, the CSAs of all four PVs were measured ~3 mm before the point at which it drains through the pulmonary venous ostium in the left atrium directly in the oblique coronal image perpendicular to the axis of the pulmonary vein. This provides the most accurate CSA, especially in cases of an oval or elongated lumen (Figure 2D). The PVI is calculated using the following formula^12^:


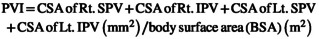


Various studies have established the reference ranges for the diameters and CSA of PVs in adult patients. The CSA of PVs used in the PVI provides important information about differences in blood flow and vascular resistance between the lungs in patients with CHD. This assists in the prediction of prognoses (Figure 2).

Significant pulmonary regurgitation (PR) can occur after the repair of TOF. Although the PR has been well tolerated for a decade or two, moderate‐to‐severe PR may eventually develop when there is significant RV dilatation and dysfunction. This requires the surgical insertion of a homograft pulmonary valve. (Table 4) RV function is best assessed by magnetic resonance imaging, 2‐D echocardiography.

**TABLE 4:** Suggested criteria for surgical pulmonary valve replacement

GEVAT (2006) based on RV regurgitant fraction RV regurgitation fraction ≥25% PLUS Two or more of the following criteria: RV end‐diastolic volume index ≥160 ml/m^2‐^ (normal values, <108 ml/m^2^) RV end‐systolic volume index ≥70 ml/m^2^ (normal values, <47 ml/m^2^) LV end‐diastolic volume index ≥65 ml/m^2^ RV ejection fraction ≤45% RV outflow tract aneurysm Clinical criteria Exercise intolerance Syncope Presence of heart failure Sustained ventricular tachycardia or^a^ QRS duration ≥ 180 ms^a^	Lee C. et al. (2012) RV end‐systolic volume index ≥80 ml/m^2^ and RV end‐diastolic volume index ≥163 ml/m^2^

^a^ Risk factor for sudden death.

## 3 SURGICAL TECHNIQUE

The operation was performed on our patient with full‐flow cardiopulmonary bypass and moderate hypothermia using repeated Del Nido crystalloid cardioplegia under general anesthesia.

**FIGURE 3:**
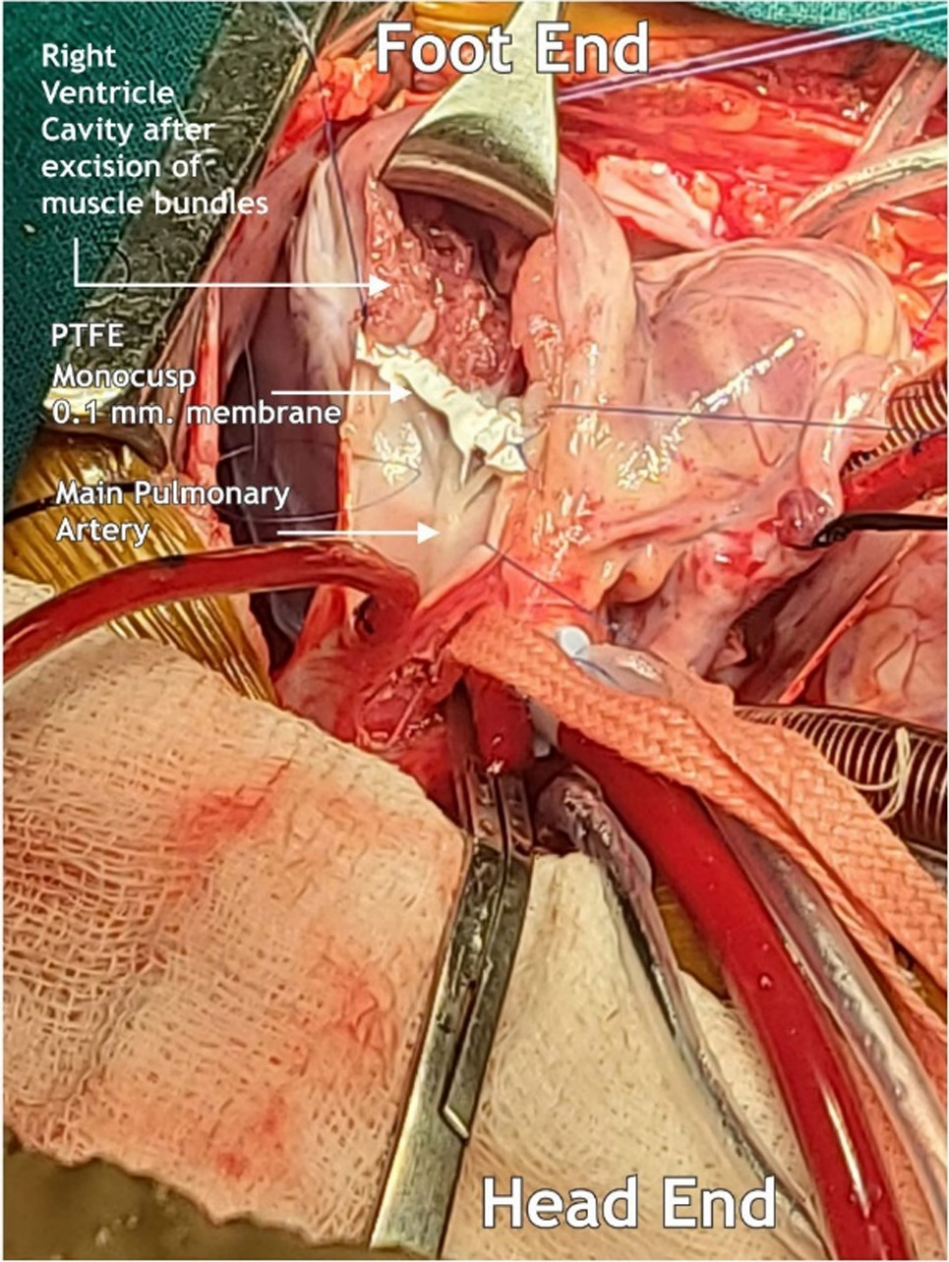
RVOT open with PTFE monocusp membrane

**FIGURE 4:**
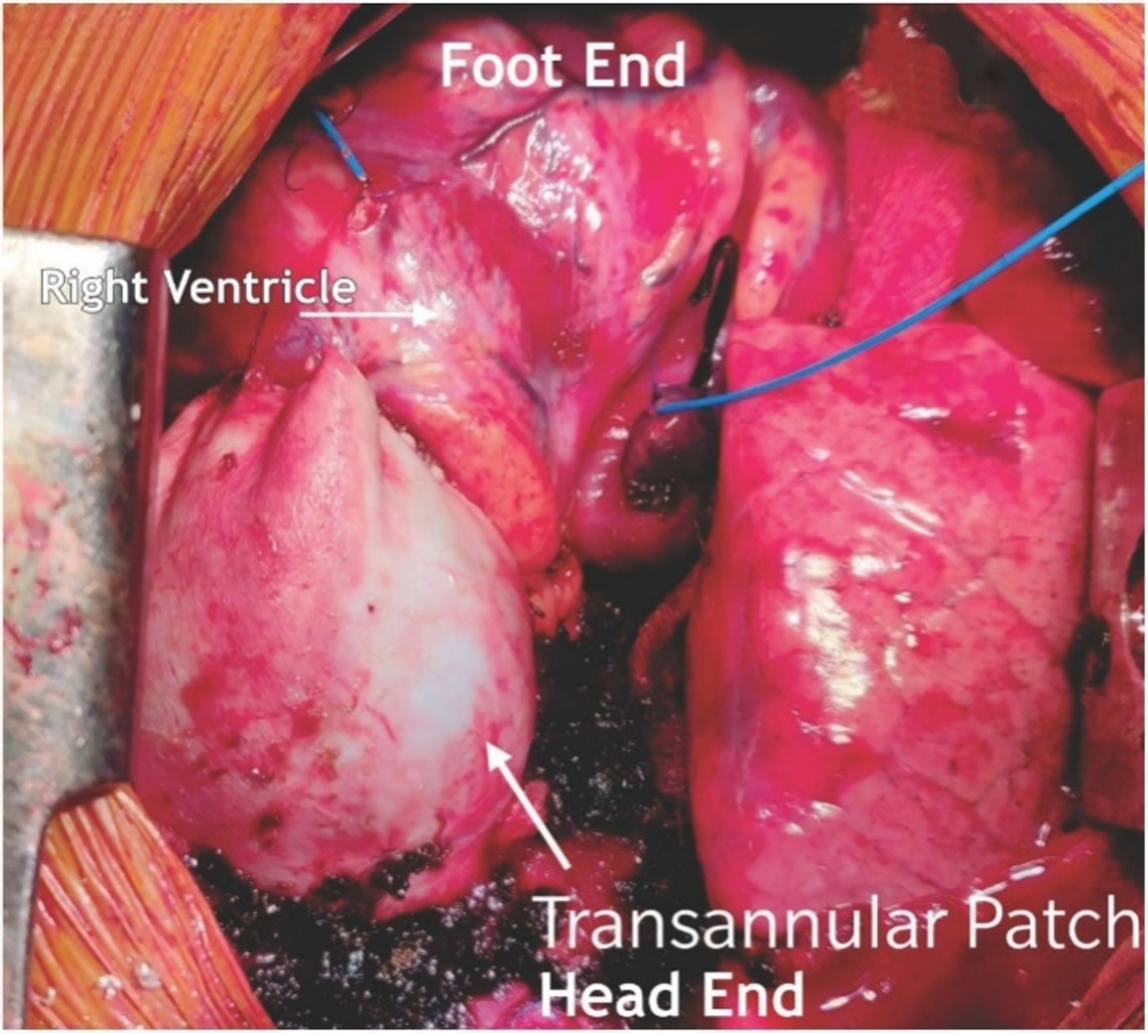
RVOT covered with autologous pericardial patch

Glutaraldehyde treated pericardial patch closure of the ventricular septal defect was performed with the continuous running of 5/0 polypropylene sutures through the right atrial (RA) approach. We then resected the right ventricular outflow tract (RVOT) via trans‐atrial and trans‐outflow approaches. We reconstructed the PTFE monocusp valve 0.1 membrane of the RVOT. We then employed TAPE augmentation (Figures 3 and 4).

## 4 MONOCUSP PREPARATION

**FIGURE 5:**
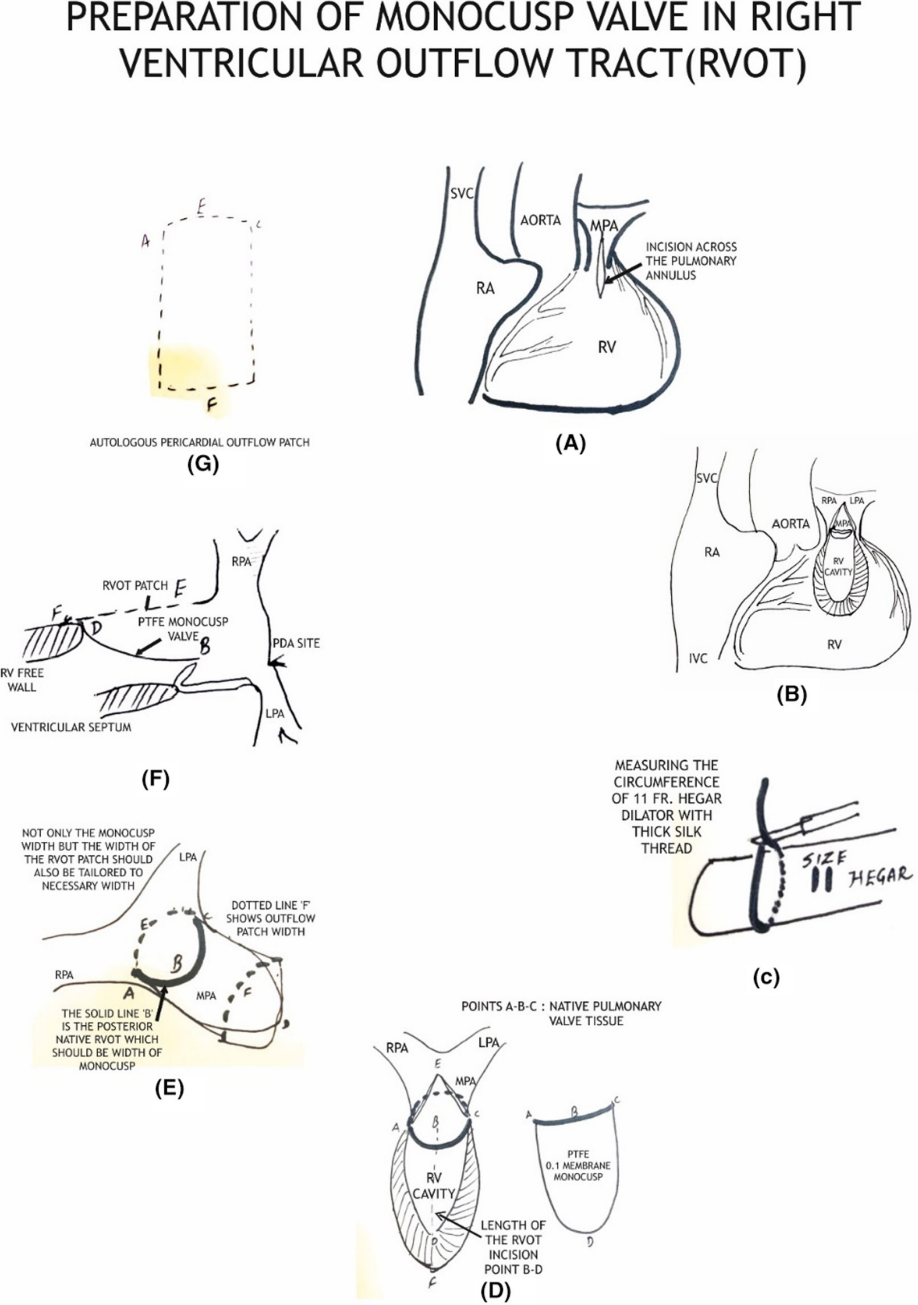
Preparation of monocusp valve in right ventricular outflow tract (RVOT)

We first measured the circumference of the RVOT from points A to C using black silk marking. A Hegar dilator two sizes larger than required for the patient's body weight was employed. We used the marked silk threads for width (points A, B, C) and the Hegar dilator to shape the membrane. Finally, we sutured the vertex of the membrane to the native area of the pulmonary valve and both the ends to the width ends of the RVOT using 6/0 polypropylene sutures. (Figure 5).

## 5 RESULTS

A molecular assay for the qualitative detection of SARS‐CoV‐2 was performed; the results of which were negative. The patient then underwent a detailed cardiological evaluation, including an electrocardiogram, a two‐dimensional (2D) echocardiogram, and a cardiac CT dynamic study.

The patient's PVI was 368.12 mm^2^/m^2^, which resulted in a smooth and uneventful postoperative course and no symptoms of low cardiac output syndrome (Tables 5 and 6).

**TABLE 5:** Postoperative data

RVOT peak gradient (mm Hg)	12 mm Hg
pRV/pLV	0.4
Ventilator hours	122 h (5 days, 2 h, 8 min)
Length of ICU stay	11 days
Hospital stay	14 days
Major complications	Nil

**TABLE 6:** Arterial blood gases done in the patient on POD 1, 3, 5, and 7 with trends in lactate and potassium (K^+^) levels

	POD 1	POD 3	POD 5	POD 7	
pH	7.50	7.43	7.44	7.46	mmHg
pCO2	35	36	42	35	mmHg
pO2	152	129	127	174	mmol/L
Na +	148	139	135	133	mmol/L
K+	3.1	3.3	3.5	4.2	mmol/L
Ca++	1.00	1.10	1.13	1.13	mmol/L
Glu	198	148	97	95	mg/dL
Lac	1.8	1.1	0.8	1.0	mmol/L
Hct	41	42	37	38	%
Ca++ (7.4)	1.04	1.11	1.15	1.16	mmol/L
HCO3‐	27.3	23.9	28.5	24.9	mmol/L
HCO3 std	28.3	24.9	28.0	26.0	mmol/L
TCO2	28.4	25.0	29.8	26.0	mmol/L
BE ecf	4.1	−0.4	4.3	1.1	mmol/L
BE (B)	4.2	−0.1	3.9	1.3	mmol/L
S02c	99	99	99	100	%
THbc	12.7	13.0	11.5	11.8	g/dL
A‐aDO2	54	40	34	−18	mmHg
pAO2	206	169	161	156	mmHg
paO2/pAO2	0.74	0.76	0.79	1.12	

Abbreviation: POD, post‐operative day.

In this study, we evaluated the predictive value of PVI for the determination of early postoperative outcomes among patients with TOF. A reduced PVI is a significant risk factor for both early mortality and prolonged postoperative recovery.^12^

Cardiac CT dynamic study is a valuable tool that can provide detailed information on the morphology of extra‐cardiac vessels, that is, the coronary vessels, PAs, aorta, and pulmonary and systemic veins.

A secondary objective of this case report was to assess the performance of polytetrafluoroethylene (PTFE) monocusp valves (MVs) (PTFE‐MVs). PTFE‐MVs do not calcify in the membrane. Instead, a well‐vascularized layer of non‐obstructive fibro‐collagenous tissue is incorporated into the PTFE with focal areas of endothelialization. The MV integrates with the RVOT patch to variable degrees. Reconstruction of the RVOT with a PTFE‐MV has proven a simple, reproducible technique with excellent early postoperative function and minimal PR. Early clinical benefits are seen in patients reconstructed with a monocusp valve compared with those who undergo transannular patch (TAP) repairs. Hence, the PVI with PTFE‐MV reconstruction of the RVOT is a valuable preoperative predictor of the early prognosis of TOF patients (Tables 7, 8, 9; Video S1).

**TABLE 7:** Minimum Acceptable Pulmonary Valve Ring Diameter (Employed by Kirklin in 1975, 1976)

Weight (kgs.)	Min.Ring Size Diameter (mm.)	Area (mm^2^)	1/2 Size
	4	13	
	5	20	
3	6	28	4
4	7	39	5
5	7.5	45	5.5
6	8	50	6
7	9	64	6.5
8	9.5	72	7
9	10	79	7.5
10	11	85	8.5
12	12	113	9
14	13	133	9.5
16	13.5	144	10
18	14	154	11
20	15	177	11
25	17	227	12
30	18.5	270	13
35	20	314	14
40	20	314	14

**TABLE 8:** Mean normal valve diameters

BSA (m^2^)	Mitral	Tricuspid	Aortic	Pulmonary
0.25	11.2	13.4	7.2	8
0.30	12.6	14.9	8.1	9
0.35	13.6	16.2	8.9	10
0.40	14.4	17.3	9.5	10
0.45	15.2	18.2	10.1	11
0.50	15.8	19.2	10.7	11
0.60	16.9	20.7	11.5	12
0.70	17.9	21.9	12.3	13
0.80	18.8	23.0	13.0	14
0.90	19.7	24.0	13.4	14
1.00	20.2	24.9	14.0	15
1.20	21.4	26.2	14.8	16
1.40	22.3	27.7	15.5	17
1.60	23.1	28.9	16.1	17
1.80	23.8	29.1	16.5	18
2.00	24.2	30.0	17.2	18

Approximate ± SD			
(0.3) = 1.9	(1.9) = 1.7	(ALL) = 1.0	(ALL) = 1.2
(0.3) = 1.6	(1.0) = 1.5		

**TABLE 9:** Prediction of postoperative residual intracardiac shunts/intravascular pressures

Sr. No.	Parameter
1	RV (or PA) systolic pressure [RVSP/PASP] = 4 (V*)^2^ + Right atrial (RA) pressure, where V* is the TR jet velocity.
2	RV (or PA) systolic pressure = Systemic SP (or arm SP) − 4(V**)^2^, where V** is the ventricular septal defect (VSD) jet velocity.
3	LVSP = 4(V***)^2^ + Systemic SP (or arm SP), where V*** is the Aortic flow velocity.

Abbreviations: LVSP, left ventricle systolic pressure; PA, pulmonary artery; RV, right ventricle; SP, systolic pressure.

## 6 DISCUSSION

There have been numerous attempts to establish useful prognostic indicators for CHD. Some indices that have shown good prognostic value include the McGoon ratio, Nakata index, pulmonary arterial index, and TNPAI. More recently, the PVI has been indicated as a predictor for early outcomes during the surgical treatment of patients with TOF. In this study, we determine the predictive value of PVI. In their study, Yuan et al. indicated that a reduced PVI is a significant risk factor for early death and prolonged postoperative recovery. They estimated the cutoff point of PVI at 300.3 mm^2^/m^2^.^12^ In our case, the PVI was 368.12 mm^2^/m^2^, which resulted in an uneventful postoperative recovery.

Pulmonary blood flow has a major effect on the prognosis of patients with CHDs, and PA parameters, such as the McGoon ratio and Nakata index, are the most commonly used parameters to evaluate pulmonary blood flow and are also frequently used as prognostic indicators for the successful surgical repair of TOF.^10^, ^12^ However, it has been shown that the PVI provides a more accurate indicator of pulmonary blood flow than that of the PAs.^10^

Another objective of this case report was to assess the performance of PTFE‐MVs. Construction of the PTFE‐MV is an inexpensive and straightforward method of creating a competent RVOT in a variety of RVOTO anomalies.^19^, ^20^ Moreover, reports have shown that PTFE‐MVs appeared to be equal or superior to biologic monocusp valves, providing perioperative RVOT competence and improved right ventricular functional characteristics,^19^ reduced ICU stay, and decreased operative morbidity and mortality in patients with TOF.^20^

Although the literature is in conclusive regarding the perioperative function and clinical benefit of PTFE‐MV for RVOT reconstruction, PTFE‐MV has proven to be a simple and reproducible technique that demonstrates excellent early postoperative function with minimal pulmonary insufficiency.^21^ All types of monocusps have been shown, particularly in the immediate postoperative period, to significantly reduce or prevent pulmonary insufficiency. The elimination of pulmonary insufficiency is associated with a faster recovery of RV function, lower central venous pressure, and less postoperative chest tube drainage.^22^ A review of 196 patients demonstrated only mild‐to‐moderate insufficiency in 58% of patients at 10 years without significant RVOT stenosis.^21^ In our study, we show that PVI with PTFE‐MV reconstruction for RVOT is a valuable preoperative predictor for the early prognosis in TOF.

The patient in our case report underwent a detailed cardiac evaluation with electrocardiogram, 2D echocardiography, and cardiac CT dynamic study. Cardiac CT is a noninvasive technique that offers several approaches to establish the hemodynamic severity of coronary artery obstructions, demonstrates a good correlation with directly measured coronary flow and fractional flow reserve,^23^ and provides incremental diagnostic value over coronary CT angiography alone for the identification of hemodynamically significant coronary artery disease.^24^

## 7 CONCLUSION

In this case report, we determined how the PVI will affect the postoperative outcome of patients with TOF who are treated surgically and whether there is a correlation between the indices and postoperative recovery. The other aspect of the study is the performance of PTFE‐MV. The PTFE‐MV does not calcify in the membrane, but rather a well‐vascularized layer of non‐obstructive fibrocollagenous tissue is incorporated within the PTFE with focal areas of endothelialization. Thus, the PTFE‐MV integrates with the RVOT patch to a variable degree.

The reconstruction of the RVOT with a PTFE MO valve has proven to be a simple and reproducible technique demonstrating excellent early postoperative function with minimal PR. Moreover, in patients reconstructed with a PTFE‐MV, there is a degree of early clinical benefit when compared with TAP repairs. Hence, PVI with PTFE‐MV for RVOT reconstruction is a valuable preoperative predictor for the early prognosis of patients with TOF. In addition, cardiac CT dynamic study is a valuable and recommended tool, as it is a noninvasive technique that can pinpoint the morphology of extra‐cardiac vessels.

## AUTHOR CONTRIBUTIONS

Vishal V. Bhende and Tanishq S. Sharma conceptualization, data curation, formal analysis, funding acquisition, investigation, methodology, project administration, resources, software, supervision, validation, visualization, writing—original draft, and writing—review and editing. Amit Kumar involved in data curation, formal analysis, investigation, methodology, project administration, software, supervision, validation, and visualization. Jigar P. Thacker involved in data curation, formal analysis, investigation, methodology, project administration, software, supervision, validation, and visualization. Deepakkumar V. Mehta, Viral B. Patel, Krishnan Ganapathy Subramaniam, Gurpreet Panesar, Kunal Soni, Kartik B. Dhami, Hardil P. Majmudar, Nirja Patel, and Sohilkhan R. Pathan contributed to data curation, formal analysis, investigation, methodology, project administration, resources, software, supervision, validation, and visualization.

## CONFLICT OF INTEREST

In compliance with the ICMJE uniform disclosure form, all authors declare the following: Payment/services info: All authors have declared that no financial support was received from any organization for the submitted work. Financial relationships: All authors have declared that they have no financial relationships at present or within the previous three years with any organizations that might have an interest in the submitted work. Other relationships: All authors have declared that there are no other relationships or activities that could appear to have influenced the submitted work.

## ETHICAL APPROVAL

Human subjects: Consent was obtained or waived by all participants in this study. Institutional Ethics Committee‐2, Bhaikaka University, Karamsad, Anand, Gujarat‐ 388,325, India, issued approval IEC/BU/2021/Cr.54/296, dated November 27, 2021. IEC received a summary of the project report titled “Higher Pulmonary Vein Index from Preoperative Computed Tomography Angiography and good Surgical Resection Ensures a Smooth Postoperative Recovery Sans low Cardiac Output Syndrome in a Tetralogy of Fallot Child ‐ Special Emphasis on Indices of Evaluation, Monocusp preparation” containing the following document[s] for review and approval toward publication/presentation: After perusal, IEC approves the project report “Higher Pulmonary Vein Index from Preoperative Computed Tomography Angiography and good Surgical Resection Ensures a Smooth Postoperative Recovery Sans low Cardiac Output Syndrome in a Tetralogy of Fallot Child ‐ Special Emphasis on Indices of Evaluation, Monocusp preparation” for presentation/publication.

## CONSENT

Written informed consent was obtained from the patient for publication of this case report and accompanying images in accordance with the journal's patient consent policy.

## Supporting information


Video S1: Post ‐ operative Bedside Echocardiography for Fallot's Tetralogy Utilizing PVI.

## Data Availability

The data that support the findings of this study are available from the corresponding author upon reasonable request.
