# Supplementary material for: Higher pulmonary vein index on computed angiography and optimum surgical resection ensures smooth postoperative recovery in Fallot's tetralogy: Special emphasis on indices of evaluation and Monocusp preparation
Source: Clin Case Rep. 2022 Jul 27;10(7):e6100. doi: 10.1002/ccr3.6100 (PMC9327839; doi:10.1002/ccr3.6100)
Supplement: Supplementary file 1 — Video S1: Post ‐ operative Bedside Echocardiography for Fallot's Tetralogy Utilizing PVI. [file CCR3-10-e6100-s001.docx]

Video 1: Post - operative Bedside Echocardiography for Fallot's Tetralogy Utilizing PVI

*Link: https://youtu.be/nHFaA7f7jAc*
